# Production of succinic acid through the fermentation of *Actinobacillus succinogenes* on the hydrolysate of Napier grass

**DOI:** 10.1186/s13068-022-02106-0

**Published:** 2022-01-18

**Authors:** Jhih-Sing Lee, Cheng-Jia Lin, Wen-Chien Lee, Hsin-Yi Teng, Meng-Hsin Chuang

**Affiliations:** Department of Chemical Engineering, Systems Biology and Tissue Engineering Research Center, National Chung Cheng University, Chiayi, Taiwan

**Keywords:** Recovery, Fermentation, Succinic acid, Lignocellulosic Biomass, Separation

## Abstract

**Background:**

Napier grass biomass can be hydrolyzed mainly containing glucose and xylose after alkaline pretreatment and enzymatic hydrolysis. This biomass can be fermented using *Actinobacillus succinogenes* to produce succinic acid. The yield of succinic acid was 0.58 g/g. Because metabolizing xylose could produce more acetic acid, this yield of succinic acid was lower than that achieved using glucose as the sole carbon source.

**Results:**

The addition of glycerol as a fermentation substrate to Napier grass hydrolysate increased the reducing power of the hydrolysate, which not only increased the production of succinic acid but also reduced the formation of undesirable acetic acid in bacterial cells. At a hydrolysate:glycerol ratio of 10:1, the succinic acid yield reached 0.65 g/g. The succinic acid yield increased to 0.88 g/g when a 1:1 ratio of hydrolysate:glycerol was used. For the recovery of succinic acid from the fermentation broth, an outside-in module of an ultrafiltration membrane was used to remove bacterial cells. Air sparging at the feed side with a flow rate of 3 L/min increased the filtration rate. When the air flow rate was increased from 0 to 3 L/min, the average filtration rate increased from 25.0 to 45.7 mL/min, which corresponds to an increase of 82.8%. The clarified fermentation broth was then electrodialized to separate succinate from other contaminated ions. After electrodialysis, the acid products were concentrated through water removal, decolorized through treatment with activated carbon, and precipitated to obtain a purified product.

**Conclusions:**

The yield of succinic acid was increased by adding glycerol to the hydrolysate of Napier grass. The downstream processing consisting of ultrafiltration membrane separation and single-stage electrodialysis was effective for product separation and purification. An overall recovery yield of 74.7% ± 4.5% and a purity of 99.4% ± 0.1% were achieved for succinic acid.

**Supplementary Information:**

The online version contains supplementary material available at 10.1186/s13068-022-02106-0.

## Introduction

Succinic acid is an intermediate metabolite of the TCA cycle and a crucial precursor that has been widely applied in fields, such as medicine, food, and macromolecule synthesis. Succinic acid can be produced through the fermentation of microorganisms, such as *Actinobacillus succinogenes* on fermentable carbon sources [1, 2]. Because of its low cost and abundance, lignocellulosic feedstock is attractive as a raw material for producing succinic acid. Cellulosic biomass is conventionally converted into fermentable sugars through pretreatment and subsequent enzymatic hydrolysis. The sugars in the resultant hydrolysate are then subjected to fermentation by succinic-acid-producing bacteria. Many renewable lignocellulose resources, such as sugar cane bagasse [3], corn fiber [4], corn stover [5, 6]*,* cotton stalk [7], corn core, rice straw, wheat straw [8], and sorghum straw [9], have been used to generate hydrolysate, which is subsequently fermented by *A. succinogenes*. In addition to the aforementioned agriculture waste, grass-based biomass has considerable potential as lignocellulosic feedstock. For example, Napier grass (*Pennisetum purpureum* Schum) grows fast and has a high biomass productivity, with its annual dry matter yield often exceeding 40 t/ha [10, 11]. Napier grass has a cellulose content of up to 40–45% dry base weight; thus, Napier grass is a potential source for renewable energy production and a potential substrate for the production of biobased chemicals, such as monosaccharides, oligosaccharides, xylitol, alcohols, and organic acids. In a previous study, we used Napier grass as a raw material for ethanol production [12]. In the current study, the hydrolysate of Napier grass was used as a low-cost feedstock for producing succinic acid.

Similar to other lignocellulosic feedstock, the hydrolysate of Napier grass mainly contains glucose and xylose, which can be used as carbon sources for microbial fermentation. Compared with xylose, *A. succinogenes* is more effective at metabolizing glucose [13, 14]. In a medium containing both glucose and xylose, the concentration and productivity of succinic acid decrease with an increase in the xylose fraction in the feed. As the percentage of xylose in a medium containing both glucose and xylose increases, the production of acetic acid and formic acid increases and the succinic acid-to-acetic acid (S/A) ratio decreases [14]. Moreover, when this bacterium is fermented on biomass hydrolysates, its main inhibitor is acetate [15]. To increase the yield of succinic acid, glycerol can be used as a carbon source together with biomass hydrolysate. Glycerol, which is a readily available and inexpensive compound, is generated during biodiesel production. The high degree of reduction per carbon makes glycerol an attractive carbon source for the production of bio-based chemicals [16].

After microbial fermentation, the fermentation broth containing organic acids must be separated and purified. Because the fermentation broth contains many substances, such as bacteria, salt, protein, and other metabolites, the separation and purification of the broth are costly processes and may even account for more than 50% of the entire production cost [17]. Therefore, a cost-effective and environmentally friendly separation and purification procedure must be developed to separate succinic acid. Many downstream separation processes, including calcium precipitation, electrodialysis, direct crystallization through acidification or using cation-exchange resins, salting-out, and reactive extraction [18, 19], have been used to separate succinic acid. Glassner and Datta developed a purification process for succinic acid that involves desalination electrodialysis and bipolar membrane electrodialysis followed by the removal of particulates from the fermentation broth [20]. A desalting electrodialysis stack consists of an alternating series of anion- and cation-selective membranes, and the water-splitting electrodialysis stack consists of alternating cation-permeable and bipolar membranes [21]. According to the review of Cheng et al. [17], a total purification yield of 60% can be achieved after two stages of electrodialysis. In the present study, a succinic acid yield of higher than 60% was achieved by conducting one-stage electrodialysis.

Succinic acid is one of the most promising platform chemical that can be derived from renewable resources. It has a wide range of applications in industry and can be made into bio-based polymers [22]. This high-value bio-based succinic acid has been extensively used in chemical, food, pharmaceutical, leather, and textile industries and can be synthesized through microbial fermentation on lignocellulosic biomass [23]. For the production of succinic acid from Napier grass biomass, this study hopes to establish a process from fermentation to downstream processing.

## Results

### Mutation of the production strain

The mutant strain Mu-B7 was selected from colonies of *A. succinogenes* treated with 3 × 10^–4^ g/L of *N*-methyl-*Nʹ*-nitro-*N*-nitrosoguanidine (NTG). At this level of NTG treatment, the survival rate was about 8%. Because the mutation rate is directly proportional to the death rate [24], we induced the bacterial mutagenesis with higher concentration of NTG, so that the death rate was high but not too high to screen for mutants. When fermentation was conducted on a 96-well plate (2 mL per well with 10 g/L glycerol and 1.8% dimethyl sulfoxide), the succinic acid yield of wild type *A. succinogenes* was 0.93 ± 0.01 g/g, while that of Mu-B7 mutant was 0.98 ± 0.01 g/g. Compared with wild type, the Mu-B7 mutant exhibited a 5% higher succinate yield. Moreover, no alcohol production was noted in the fermentation of the Mu-B7 mutant. Therefore, on the basis of the metabolic pathway, we inferred that the Mu-B7 mutant may have been genetically altered on alcohol dehydrogenase. Consequently, we designed polymerase chain reaction primers to amplify and sequence the gene of alcohol dehydrogenase (Asuc_0403). As shown in Additional file 1: Fig. S1, the DNA sequence alignment results indicated that the 100th C was mutated to A, which corresponds to a change in the 34th amino acid of alcohol dehydrogenase from leucine to methionine. The relationship between this amino acid mutation and activity of alcohol dehydrogenase needs to be further explored. In summary, to increase the yield of succinic acid from glycerol by *A. succinogenes*, we used chemical mutagenesis and glycerol-containing agar plate screening, and it was found that the mutant strain reduced the formation of by-product ethanol.

### Fermentation on the hydrolysate of pretreated Napier grass

**Fig. 1:**
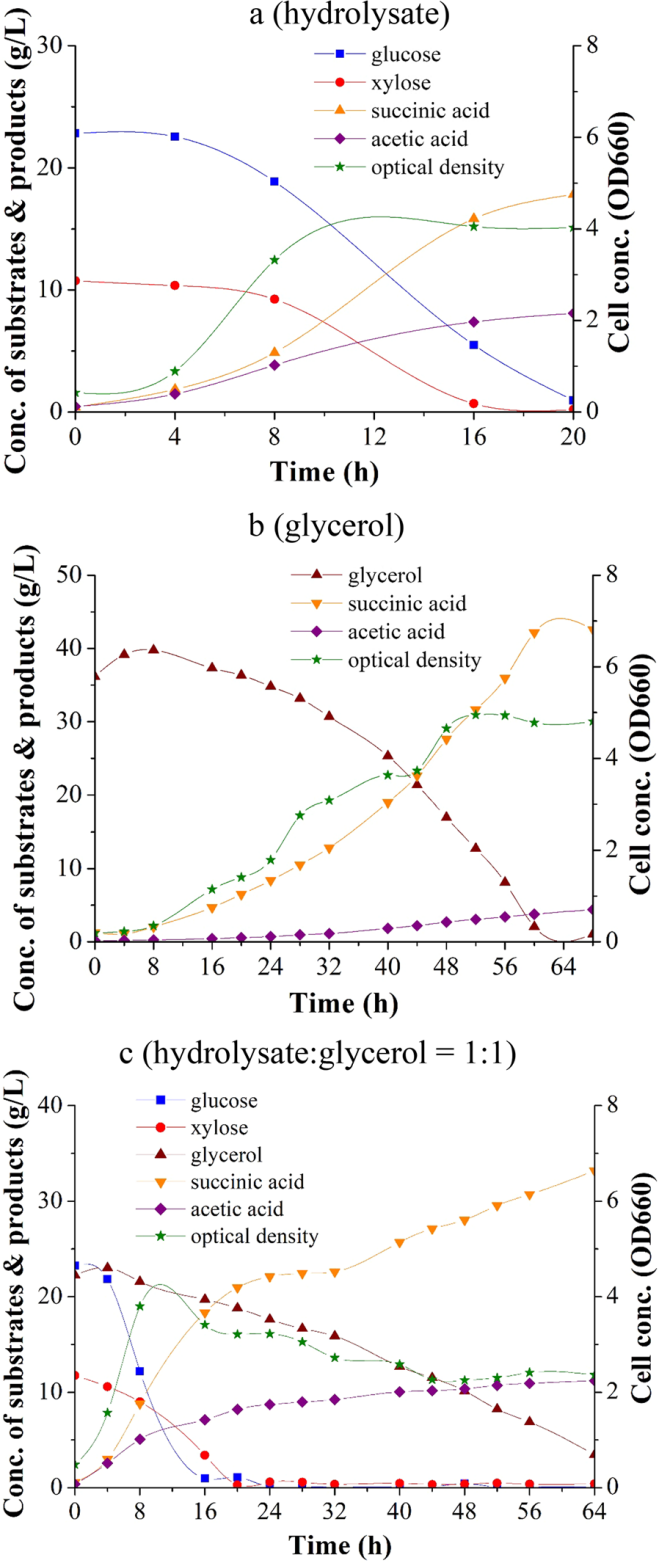
Results obtained for the fermentation of succinic acid on **a** hydrolysate of Napier grass, **b** glycerol, and **c** mixture with a 1:1 mass ratio of the hydrolysate of Napier grass and glycerol

The hydrolysis of NaOH-pretreated Napier grass by enzymatic saccharification resulted in a sugar mixture comprising 50.63 ± 5.06 g/L of glucose and 8.92 ± 1.40 g/L of xylose. These data are the average and standard deviation of the results of 5 repeated runs. However, the hydrolysate was sterilized in an autoclave at 121 °C for 30 min before being mixed with the fermentation medium for the production of succinic acid. The soluble xylose oligomers in the hydrolysate could be further hydrolyzed to xylose, so the ratio of xylose to glucose at the beginning of the fermentation was higher than the composition of the just made hydrolysate. Figure 1 illustrates the fermentation on the hydrolysate of Napier grass. This hydrolysate was used as the carbon source for the fermentation. Because the main components of the aforementioned hydrolysate were glucose and xylose, bacteria could quickly enter the log phase of growth and grow rapidly on the hydrolysate. Glucose and xylose were consumed simultaneously; however, since the starting concentration of xylose was lower than glucose, xylose was completely consumed earlier than glucose. After approximately 20 h, the carbon source was almost exhausted, and only less than 5% of the initial value was left. In addition to succinic acid, acetic acid was a major by-product of the fermentation process. The quantity of the formed acetic acid was approximately half that of the formed succinic acid. Repeated runs resulted in an average succinate yield and average productivity of 0.58 ± 0.01 g/g and 0.79 ± 0.07 g/L/h, respectively (Table 1). The yields and productivity of succinic acid obtained in this study in the fermentation on the hydrolysate of Napier grass are similar to those obtained in [7, 8, 25] in the fermentation on the hydrolysates of other lignocellulosic feedstocks. The yield and productivity of succinate reported in the literature [7, 8, 25] range from 0.58 to 0.66 g/g (succinate yield of 0.58 g/g from corn cob [25], 0.66 g/g from corn stalk [7], and 0.63 g/g from rice straw [8]) and from 0.37 to 0.79 g/L h, respectively.

**Table 1:** Results obtained for the fermentation of succinic acid on hydrolysate with the addition of glycerol at different hydrolysate:glycerol ratios

Carbon source	Hydrolysate	10:1	5:1	2:1	1:1	Glycerol	Glucose	Xylose
Titer of SA (g/L)	17.54 ± 3.80	20.23 ± 2.31	20.36 ± 0.37	22.56 ± 1.15	30.76 ± 3.44	42.30 ± 0.40	25.72 ± 1.5	21.10 ± 0.72
SA yield (g/g)	0.58 ± 0.01	0.65 ± 0.00	0.65 ± 0.01	0.70 ± 0.04	0.88 ± 0.01	1.15 ± 0.04	0.73 ± 0.03	0.57 ± 0.04
AA yield (g/g)	0.29 ± 0.03	0.29 ± 0.01	0.27 ± 0.01	0.31 ± 0.04	0.31 ± 0.01	0.13 ± 0.01	0.21 ± 0.02	0.24 ± 0.06
S/A	2.00 ± 0.07	2.30 ± 0.07	2.34 ± 0.06	2.25 ± 0.23	2.82 ± 0.21	9.81 ± 0.22	5.01 ± 2.38	2.41 ± 0.40
Productivity (g/L/h)	0.79 ± 0.07	0.84 ± 0.10	0.73 ± 0.01	0.49 ± 0.06	0.53 ± 0.01	0.61 ± 0.04	1.08 ± 0.06	0.76 ± 0.02

*SA* succinic acid, *AA* acetic acid, *S/A* the ratio of succinic acid to acetic acid

### Fermentation on glucose, xylose, and glycerol

Whether it is glucose or xylose *A. succinogenes* can easily ferment it to produce succinic acid. When using glucose as the carbon source for fermentation, bacteria quickly entered the log phase of growth and grew rapidly (Additional file 1: Fig. S2). The final fermentation time was 24 h, and the amount of succinic acid obtained was 25.72 ± 1.48 g/L. Moreover, the yield of succinic acid, the yield of acetic acid, the S/A ratio, and the productivity of succinic acid were calculated to be 0.73 ± 0.03 g/g, 0.21 ± 0.02 g/g, 5.01 ± 2.38, and 1.08 ± 0.06 g/L h, respectively (Table 1).

Compared with glucose, fermentation with xylose as a carbon source resulted in a slower growth of bacteria and a later entry into the logarithmic phase. When using xylose as the carbon source for fermentation, the final fermentation time was 28 h and the amount of succinic acid extracted was 21.10 ± 0.72 g/L. Moreover, the yield of succinic acid, the yield of acetic acid, the S/A ratio, and the productivity of succinic acid were calculated to be 0.57 ± 0.04 g/g, 0.24 ± 0.06 g/g, 2.41 ± 0.40, and 0.76 ± 0.02 g/L h, respectively. The yield of acetic acid was marginally higher when using xylose as the carbon source for fermentation than when using glucose. This result was obtained, because a higher quantity of adenosine triphosphate (ATP) was consumed when using xylose as the carbon source for fermentation than when using glucose. Thus, bacterial cells had to produce additional acetic acid to increase the quantity of ATP for ensuring the balance of energy use when xylose was adopted as the carbon source. This phenomenon in which ATP required for xylose metabolism is compensated by the formation of acetate is also found in *Escherichia coli* [26]. The hydrolysate of Napier grass contains both glucose and xylose. In this study, the yield of succinic acid (0.58 g/g) when the hydrolysate was used as the carbon source was significantly less than the yield when glucose was used as the carbon source (0.73 g/g).

The time course of the fermentation of succinic acid on glycerol are illustrated in Fig. 1. When glycerol was used as the substrate (carbon source), the bacteria grew slowly, so the lag period was longer compared to fermentation on glucose or xylose. However, after entering the log phase of growth, the bacteria began to grow rapidly. Batch fermentation was completed in 68 h when using glycerol as the carbon source. Although the fermentation time was long when using glycerol as the carbon source, the yield of succinic acid was considerably higher when using glycerol as the carbon source than when using saccharides, such as glucose or xylose as the carbon source. A high succinic yield of 1.15 ± 0.04 g/g was achieved with glycerol. Moreover, the fermentation of glycerol produced a relatively low quantity of acetic acid, and the S/A value was as high as 9.81 ± 0.22 when using glycerol as the carbon source.

### Addition of glycerol to the hydrolysate of Napier grass

When glycerol was added to the hydrolysate of Napier grass for the fermentation, the glucose and xylose in the hydrolysate were metabolized first; thus, the succinic acid content initially originated from these two monosaccharides in the hydrolysate. When the aforementioned monosaccharides were rapidly depleted, microorganisms slowly metabolized glycerol to produce succinic acid. When the hydrolysate-to-glycerol ratio (in terms of the glucose in the hydrolysate and glycerol) decreased, the fermentation time increased (Fig. 1c and Additional file 1: Fig. S2). In the study of engineered *Yarrowia lipolytica* co-fermenting xylose and glycerol to produce succinic acid in shake flasks, it was found that the consumption of xylose slowed down, indicating a sign of catabolite inhibition [27]. This phenomenon did not appear in the fermentation of *A. succinogenes* on the combined use of hydrolysate (containing xylose) and glycerol.

The yield of succinic acid was 0.58 ± 0.01 g/g when the hydrolysate of Napier grass was used as the carbon source. The yield increased with an increase in the quantity of glycerol added. The highest yield of succinic acid (i.e., 0.88 ± 0.01 g/g) was achieved when the hydrolysate:glycerol ratio was 1:1. The yield of succinic acid and the S/A ratio increased by up to 52% and 41%, respectively. This increase in the succinate yield was caused by the metabolism of glycerol. The addition of glycerol resulted in an increase in the yield of succinic acid; however, beyond a certain level, the addition of glycerol caused a decrease in the productivity of succinic acid. The productivity of succinic acid increased from 0.79 ± 0.07 g/L h when using only the produced hydrolysate as the carbon source to 0.84 ± 0.10 g/L h when adding glycerol at a hydrolysate:glycerol ratio of 10:1 to the fermentation broth. Below this ratio, the productivity of succinic acid decreased as the proportion of glycerol increased because of an increase in the fermentation time. The productivity of succinic acid at hydrolysate:glycerol ratios lower than 10:1 was lower than that when glycerol was not added.

Because a relatively small quantity of acetic acid was produced through glycerol metabolism, the S/A ratio was high when glycerol was used as the carbon source. The S/A ratio increased marginally from 2.00 ± 0.07 to 2.82 ± 0.21 when the hydrolysate:glycerol ratio was 1:1 (Table 1). Because the glucose-to-xylose ratio was fixed in the hydrolysate, the yield of acetic acid was maintained at approximately 0.3 g/g.

### Recovery and purification of succinic acid

**Fig. 2:**
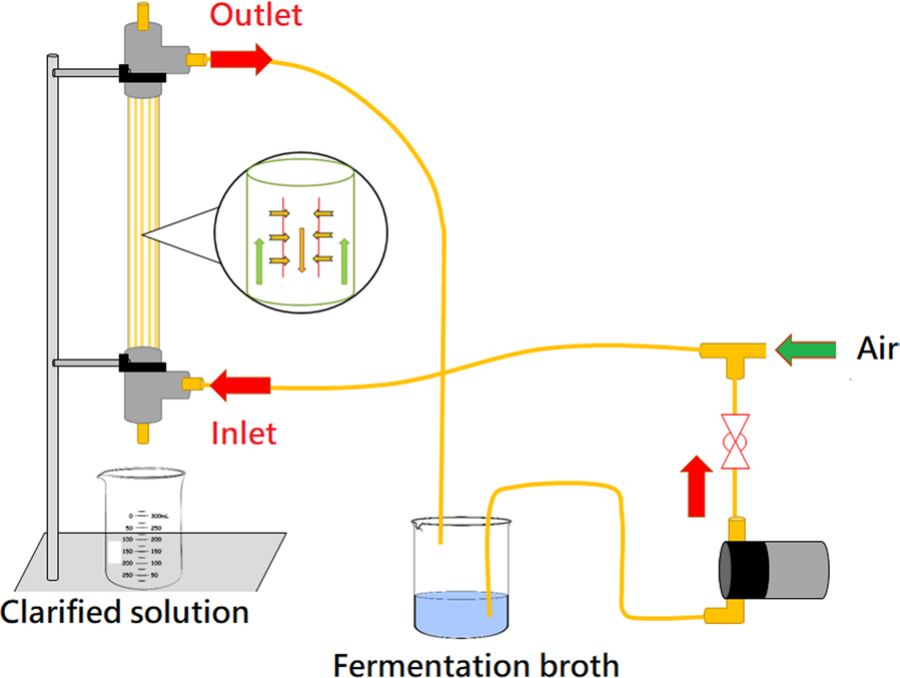
Setup for the separation of bacterial cells from the fermentation broth

**Fig. 3:**
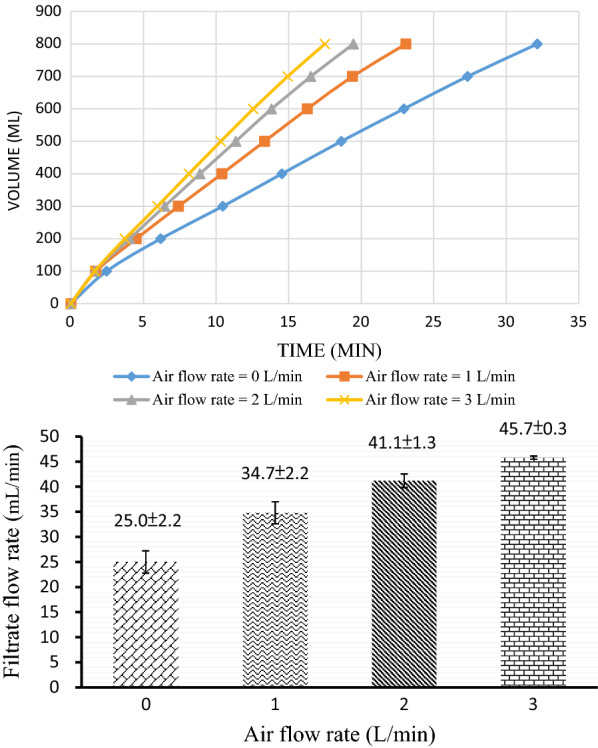
Influence of the gas flow rate on the membrane microfiltration of the fermentation broth. The volume of the filtrate changed with time (upper panel), and the filtration rate depended on the flow rate of air sparging (lower panel)

**Fig. 4:**
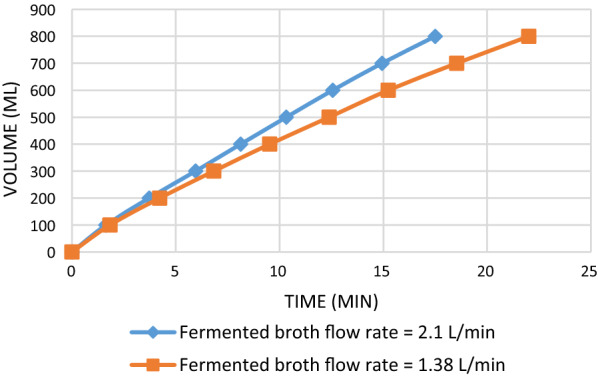
Influence of the liquid flow rate on the membrane microfiltration of the fermentation broth

For the recovery of succinic acid from the fermentation broth, an outside-in module of an ultrafiltration membrane (Fig. 2) was used to remove bacterial cells effectively. It is a crossflow membrane filtration system. The purging air in the feed side enhanced the filtration rate, defined as the volume of filtrate obtained per unit time. The filtration speed increased as the aeration flow rate increased. As displayed in Fig. 3, the filtration of 800 mL of broth required 32.1 min without gas sparging. When the volumetric flow rate of the fermentation broth was fixed at 2.1 L/min and the gas sparging rate was 3 L/min, the time required to complete the filtration process was reduced to 17.5 min. The filtration rate gradually increased with an increase in the air flow rate (the outlet pressure of the air compressor was fixed at 1 kg/cm^2^). When the air flow rate was increased from 0 to 3 L/min, the average filtration rate increased from 25.0 ± 2.2 to 45.7 ± 0.3 mL/min, which corresponds to an increase of 82.8%. As the same air flow rate of 3 L/min, reducing the feed (fermentation broth) flow rate from 2.1 to 1.38 L/min caused the decrease in the filtration rate from 45.7 ± 0.3 to 36.4 ± 1.0 mL/min. Moreover, the time required to complete the filtration of 800 mL of broth increased as the feed rate decreased (Fig. 4).

**Fig. 5:**
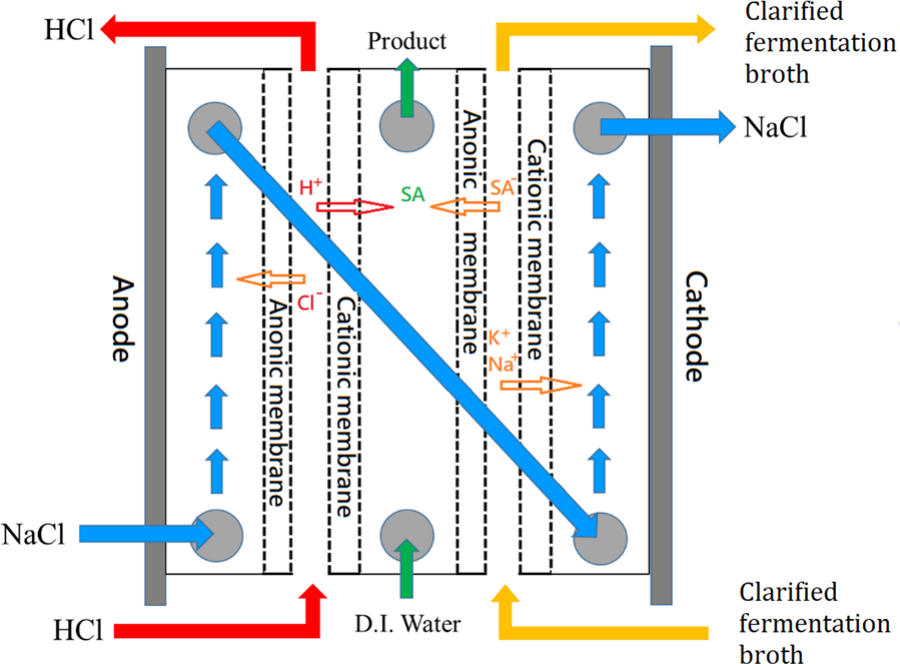
Schematic of the electrodialysis setup for the purification of succinic acid

The clarified broth was electrodialized using an electrodialysis equipment as shown in Fig. 5 to separate the succinate from other contaminated ions. Under the influence of an applied electric potential difference, the succinate and cationic ions in the clarified fermentation broth can permeate through the anionic membrane and the cationic membrane, respectively. In the intermediate circulation channel, succinate meets with protons (passing through another cationic membrane) from HCl to form succinic acid. The separated cations such as Na+ and K+ meet with the chloride ion splitted from HCl to form salts in a circulating flow channel. Electrodialysis was conducted for 28 h on the clarified broth at a volumetric flow rate of 30 mL/min using a 10-V direct-current power source and a maximum current of 3.17 A. In an example trial, the original concentration of succinate was 25.91 g/L in 1.8 L of the fermentation broth. After 28 h of electrodialysis, the concentration of succinic acid decreased to 0.86 g/L and the volume of the fermentation broth decreased to 1.32 L. Most of the succinate was added into DI water to form succinic acid, and the concentration of succinic acid in the product stream reached 37.71 g/L. After electrodialysis, the recovery rate of succinic acid was 92% ± 2% in three tests (Table 2).

**Table 2:** Results obtained for succinic acid recovery and purification (*n* = 3)

Recovery rate after electrodialysis (%)	92 ± 2
Recovery rate after concentration and decolorization (%)	81.3 ± 4.2
Total recovery rate (%)	74.7 ± 4.5
Purity (%)	99.4 ± 0.1

After electrodialysis, the acid products were subjected to concentration through water removal, decolorization through treatment with activated carbon, and precipitation to yield purified organic acids. The succinic acid solution obtained after electrodialysis was heated to 100 °C to eliminate its water content. When the concentration of this solution reached 400 g/L, the solution was cooled to 10 °C to produce precipitated crystals, which were then dissolved in DI water. Decolorization was conducted by adding 1% (w/v) activated carbon to the succinic acid solution at a temperature of 75 °C. The recovery rate of succinic acid after decolorization treatment was 81.3% ± 4.2%. According to the amounts of succinic acid obtained after electrodialysis, solution concentration, and decolorization, the total recovery yield of succinic acid was calculated to be 74.7% ± 4.5%. The purity of the final succinic acid product was 99.4% ± 0.1%. The purity of succinic acid was determined by quantifying the mass of succinic acid in the purified product (dried crystals) by HPLC.

## Discussion

In this study, we used Napier grass as the biomass source. After alkali pretreatment, Napier grass was subjected to hydrolysis with cellulosic enzymes to produce a hydrolysate rich in glucose and xylose. This hydrolysate was then used as the carbon source for the production of succinic acid. However, the succinic acid yield was lower when using the aforementioned hydrolysate as the carbon source than when using glucose or glycerol as the carbon source. The succinic acid yield and productivity decreased with an increase in the xylose fraction in the feed. This result is consistent with those in the literature for batch and continuous fermentation [13, 14]. Moreover, the yield of acetic acid reached 0.29 ± 0.03 (g/g), which resulted in a low S/A ratio of 2.00 ± 0.07. Compared with glucose and xylose, glycerol can generate a stronger reducing power [16]. Our aim was to add glycerol to the fermentation medium to increase the yield of succinic acid and reduce the production of acetic acid. The results indicate that the addition of glycerol to the fermentation medium increased the yield of succinic acid but did not reduce the yield of acetic acid. Moreover, the fermentation time increased and thus the productivity of succinic acid decreased as the proportion of glycerol in the fermentation medium increased. When the hydrolysate:glycerol ratio was 10:1, the productivity of succinic acid improved by 6%. The productivity of succinic acid increased from 0.79 ± 0.07 g/L h when using only the produced hydrolysate as the carbon source to 0.84 ± 0.10 g/L h when adding high-reducing-power glycerol at a hydrolysate:glycerol ratio of 10:1 to the fermentation broth. This increase in the productivity of succinic acid can be attributed to the increased yield in succinic acid caused by the addition of glycerol; however, this increase in yield is offset by a longer fermentation time.

The balance of NAD^+^/NADH is a crucial factor that affects the yield of succinic acid. As shown in the metabolic pathways of *A. succinogenes* [28], malate dehydrogenase uses NADH to reduce oxaloacetate to l-malate on the way from phosphoenolpyruvate to succinate. On the other hand, the pyruvate dehydrogenase complex catalyzes the conversion of pyruvate to acetyl-CoA, yielding NADH, and CO_2_. When microorganisms produce succinate, a large quantity of NADH is consumed; however, the production of acetic acid via acetyl-CoA can accompany the production of NADH. Consequently, acetic acid is produced during the production of succinic acid. In addition, if the carbon source contains xylose, the yield of acetic acid is high. This result can be inferred from the metabolic pathways of *A. succinogenes* [28]. The metabolization of xylose requires the consumption of one ATP molecule, whereas the metabolization of acetic acid can generate one ATP molecule [28]. A considerably higher quantity of ATP was consumed when xylose was used as the carbon source than when glucose or glycerol was used as the carbon source. Acetic acid was generated to produce the additional ATP required to metabolize xylose. Because of the presence of xylose, the formation of acetic acid could not be reduced even with the addition of glycerol. Finally, compared with the fermentation on xylose, the fermentation on the hydrolysate resulted in a marginal increase in acetic acid production. This result suggests that in addition to xylose, some unknown inhibitors in the hydrolysate may have caused the formation of acetic acid. According to the literature [29], the potential inhibitors in the hydrolysate include furfural and hydroxymethylfurfural (HMF). These inhibitors cause a decrease in the intracellular ATP concentration. In addition to inhibiting growth, it also inhibits the xylose consumption [30]. Neutralizing the effects of these toxic substances requires extra ATP from the cell, which leads to more acetic acid production. The addition of glycerol into the hydrolysate of Napier grass did not reduce the formation of acetic acid but increased the succinic acid yield. The yield and productivity of succinic acid obtained in this study compare favorably with those obtained for fermentation in different types of hydrolysates in other studies [7, 8, 22].

For the recovery and purification of succinic acid, membrane ultrafiltration was conducted to remove bacterial cells from the fermentation broth. The clarified broth was then further purified through one-stage electrodialysis. Because hollow-fiber PVDF membranes with a nominal pore size of 0.05 µm were used in the outside-in module of the adopted ultrafiltration system, bacterial cells could be completely removed from the fermentation broth. The results indicated that if an air flow rate of 3 L/min was present at the shell side, the filtration rate could be increased by 82.8%. A filtration rate of 45.7 mL/min corresponds to a permeate flux of 27.4 L/m^2^ h. This permeate flux is close to that observed in the ultrafiltration of fermented cheese whey broth when using a membrane with a molecular weight cutoff of 20,000 Da [31]. Cells of *Bifidobacteria longum* and most proteins can be retained in and separated from lactic acid in a fermentation broth through cross-flow membrane ultrafiltration. Similarly, the membrane ultrafiltration process performed in this study can effectively separate cells of *A. succinogenes* and most proteins from succinic acid.

The fermentation broth was circulated outside the fibers, and permeate was collected from the lumen of the fibers. This outside-in flow configuration allowed efficient air sparging, which enhanced particle removal and increased the recovery rates. The gas sparging process involved injecting gas with the feed during filtration [32]. Our results indicate that the flux of the permeate increased considerably when air sparging was conducted at the feed side. This finding is consistent with the finding of previous studies that the introduction of gas–liquid two-phase flow significantly improves the performance of certain membrane processes [33]. When the air flow rate increased in the current study, the filtration rate increased and the required filtration time decreased. The presumed reason for the aforementioned result is that when the fermentation broth entered the membrane group with air, the flow rate of the fermentation broth across the membrane surface increased because of the inflow of air, hampering the accumulation of bacteria on the membrane. A reduction in the clogging of pores on the membrane surface increased the filtration rate and reduced the filtration time. Moreover, when the feed flow rate of the fermentation broth decreased, the required filtration time gradually increased and the volumetric flow rate of the filtrate decreased. These results were obtained, because under the same air sparging conditions, when the amount of feed in the fermentation broth is reduced, air bubbles occupy an excessive membrane surface area, which increases the filtration time and reduces the volume flow of the filtrate.

Electrodialysis can be used as the main step in the purification of succinic acid. The electrodialysis setup used in this study, which comprised two anionic and two cationic membranes, was effective for the primary purification of succinic acid. Two cationic and two anionic membranes were arranged alternately in sequence between the cathode and the anode. During electrodialysis, the succinate in the fermentation broth and the protons of low-concentration HCl penetrated the anionic and cationic membranes, respectively, to form succinic acid in the central channel. The cations in the fermentation broth and chloride penetrated the cationic and anionic membranes to form salts. Desalting and the formation of succinic acid from succinate were achieved through single-stage electrodialysis. The succinic acid yield achieved in this study through single-stage electrodialysis (92%) is considerably higher than those achieved in previous studies through two-stage electrodialysis (60%) [17, 18, 20]. Electrodialysis is an effective method for the removal of impurities from clarified fermentation broth. Single-stage electrodialysis with a simple setup is cost-effective for achieving a high recovery yield for succinic acid.

In summary, an electrodialysis setup comprising four circulation channels can not only provide high-purity succinic acid but also fully utilize the fermentation broth as an electrolyte salt. The salts produced through electrodialysis can also be reused for the preparation of fermentation media. Electrodialysis can be combined with solution concentration through water removal, decolorization through treatment with activated carbon, and precipitation to develop a biological process for producing succinic acid in industrial applications.

## Conclusions

After pretreatment and enzymatic hydrolysis, sugars from Napier grass biomass can be fermented by *A. succinogenes* to produce succinic acid. The addition of glycerol to the hydrolysate of Napier grass caused a significant increase in the yield of succinic acid. The highest yield of succinic acid (0.88 g/g) was obtained when the hydrolysate:glycerol ratio was 1:1. The yield of succinic acid and the S/A ratio increased by up to 52% and 41%, respectively. The highest productivity of succinic acid (0.84 g/L h) was obtained at a hydrolysate:glycerol ratio of 10:1. Because of the presence of xylose in the hydrolysate of Napier grass, the S/A ratio could not be increased considerably. A high quantity of acetic acid was produced by microorganisms to compensate for the lack of energy when xylose was used as the carbon source. However, the addition of glycerol compensated for this deficiency. Glycerol and the hydrolysate of Napier grass biomass could be effectively used together in the production of succinic acid through *A. succinogenes* fermentation.

After fermentation, bacterial cells were separated from the fermentation broth using an outside-in module of an ultrafiltration system and conducting air sparging at the feed side. The flux of the filtrate was considerably enhanced by conducting air sparging at the feed side. Air sparging at a gas flow rate of 3 L/min increased the filtration rate by 82.8%. Succinic acid was purified from the clarified fermentation broth through single-stage electrodialysis by adopting a setup containing four circulating channels. Single-stage electrodialysis enabled the desalination of the succinate in the clarified broth and the formation of succinic acid with a yield rate of 92% from the desalinized succinate. This yield rate is considerably higher than those achieved using two-stage electrodialysis in previous studies. The succinic acid product obtained after electrodialysis was concentrated through water removal, decolorized through activated carbon treatment, and precipitated to obtain pure succinic acid with a high recovery rate. The proposed process of succinic acid production, including the downstream separation and purification of succinic acid after the fermentation of *A. succinogenes* on the hydrolysate of Napier grass and glycerol, is suitable for industrial application.

## Methods

### Strain mutation

The mutation of *A. succinogenes* 130Z (BCRC 80310) was induced using NTG. For achieving mutation, grown bacterial cells of *A. succinogenes* were mixed with 0.3 mL NTG (0.001 g/L) and 0.7 mL phosphate buffer solution (0.1 M, pH 7) and then allowed to incubate at 37 °C and 175 rpm for 30 min. The bacterial culture was washed twice with phosphate buffer, diluted by 10^6^ to 10^7^ times, and spread on an agar plate containing tryptic soy broth (TSB) medium, which was supplemented with 15 g/L of agar, 3 g/L of NaH_2_PO_4_⋅2H_2_O, 3.17 g/L of Na_2_HPO_4_, 2.5 g/L of glycerol and 1.8% (v/v) of dimethyl sulfoxide (DMSO). The TSB medium contained 17 g bactotryptone, 3 g bactosoytone, 2.5 g bactodextrose, 5 g sodium chloride, and 2.5 g dipotassium phosphate per liter. Single bacterial colonies were extracted and placed into a 96-well plate containing 100 μL of TSB medium. This plate was incubated at 37 °C and 175 rpm in the presence of CO_2_. The mutant strain with the highest succinate yield was denoted as Mu-B7.

### Preparation of hydrolysate

For alkaline pretreatment, 500 g of Napier grass was soaked in 10% NaOH at a ratio of 1:20 (w/v) and a temperature of 90 °C for 1 h. The pretreated product was then washed until aqueous filtrate reached an appropriate pH range (7.0–8.5) using running water. For enzyme hydrolysis, the pretreated product, which comprised 10% water-insoluble-solids (WIS), was mixed with citric acid buffer (0.05 M, pH = 4.8). After autoclaving at 121 °C for 30 min, the enzyme Novozymes Cellic^®^ CTec2 (0.08 mL/g WIS) and Tween-80 (0.01 g/g WIS) were added to the aforementioned mixture. The resultant mixture was then incubated at 50 °C for 96 h. Finally, the supernatant obtained after centrifugation at 4800×*g* for 30 min was the hydrolysate of Napier grass. Glucose and xylose concentrations in the hydrolysate were assayed by HPLC.

### Succinic acid fermentation

*Actinobacillus succinogenes* was cultured in a TSB medium to obtain inoculum. A total of 2 mL of inoculum was used to inoculate 80 mL of an intermediate medium at 37 °C. The resultant solution was shaken at 175 rpm under 0.5 vvm of CO_2_ for 24 h. The intermediate medium was composed of 5 g/L of yeast extract, 10 g/L of NaHCO_3_, 8.5 g/L of NaH_2_PO_4_·2H_2_O, 15.5 g/L of K_2_HPO_4_, 5 g/L of glucose, and 5 g/L of glycerol. A total of 80 mL of grown culture was then inoculated into a 5-L benchtop fermenter (FB-6S, Firstek Scientific Co., Taiwan) containing 800 mL of fermentation medium. The fermentation medium comprised 10 g/L of yeast extract, 10 g/L of NaHCO_3_, 15.5 g/L of NaH_2_PO_4_·2H_2_O, 6.8 g/L of K_2_HPO_4_, 1 g/L of NaCl, 0.05 g/L of MgSO_4_, and 50 g/L carbon source (glucose, xylose, glycerol, or the hydrolysate). If the carbon source was glycerol or glycerol combined with the hydrolysate of Napier grass, 17% dimethyl sulfoxide was added. Because of the high degree of reduction per carbon, glycerol has a distinct advantage over oxidized carbohydrate-based feedstocks, such as glucose and xylose [16]. Thus, we propose the addition of glycerol into the fermentation medium to increase the yield of succinic acid and reduce the production of acetic acid. To prepare the fermentation medium, 400 mL of nutrient components without carbon sources and 400 mL of sterilized hydrolysate were mixed. Glycerol was added externally, with different hydrolyzate:glycerol ratios. The ratio of hydrolysate:glycerol was based on the amount of glucose in the hydrolysate and the amount of additional glycerol.

The fermentation of succinic acid was conducted at a temperature of 37 °C, a pH of 6.7, and a rotation speed of 200 rpm under 0.5 vvm of CO_2_. The pH was controlled to 6.7 using 2 N sodium hydroxide as the regulator for the first 7 h of the fermentation and then using 8.3 N magnesium hydroxide as the regulator for the remaining fermentation time. Samples were collected every 4 h to determine the concentrations of cells (OD660), substrates, and products in the fermentation broth. The fermentation was stopped when the carbon source was consumed until below 5% of the initial value. The calculation of productivity was based on the amount of product and fermentation time, while the calculation of yield was based on the grams of consumed substrate (all carbon sources) and generated product. Data in Table 1 was collected from 2 to 3 replicates of experimental runs.

### Recovery and purification of succinic acid

For the recovery of succinic acid from the fermentation broth, an outside-in module of an ultrafiltration membrane was used to remove bacterial cells (Fig. 2). This membrane module was made from a hollow-fiber polyvinylidene difluoride (PVDF) membrane with a nominal pore size of 0.05 µm and a total membrane area of 0.1 m^2^. As displayed in Fig. 2, the fermentation broth was pumped to meet the air at a three-way valve. This air originated from an air compressor and passed through a float flow meter with an adjustable intake rate. When the pump and air compressor supplied the fermentation liquid and air simultaneously, the fermentation liquid and air flowed out of the three-way valve and entered the membrane filtration module. Under gas sparging conditions, the flow direction of the fermentation broth was parallel to the membrane surface. Cells and particulate matter were blocked outside the membrane, and the remainder of the fermentation broth passed through the membrane to become filtrate. The cross-flow filtrate then flowed to the collection tank. The fermentation broth that did not pass through the membrane was returned with air from the membrane module to the feed tank, as displayed in Fig. 2. The volumetric flow rate of fermentation was adjusted using a ball valve. During filtration, the time required to filter each 100 mL of filtrate was recorded to calculate the volume flow rate of the filtrate.

The clarified broth was then electrodialized using an electrodialysis equipment (Fig. 5), provided by Shell-Kwong-Sir Enterprise Co., Ltd. (Tainan, Taiwan), to separate succinate from other contaminated ions. Two anionic and two cationic membranes were installed alternately in sequence between the anode and the cathode. Moreover, four circulation channels were established for different aqueous solutions. The clarified fermentation broth and 0.5 M hydrochloric acid were passed through two side channels located between the anionic and cationic membranes. During electrodialysis, succinate and protons from both the clarified fermentation broth and HCl passed through the anionic and cationic membranes, respectively, and met at the central channel, in which distilled (DI) water was circulated, to form succinic acid. To maintain electrical conductivity, the channels between the two electrodes and the ionic membrane were connected and an aqueous NaCl solution with a conductivity of 2 mS/cm was circulated in them. Electrodialysis was conducted for 28 h on the clarified broth at a volumetric flow rate of 30 mL/min using a 10-V power source and a maximum current of 3.17 A.

The solution obtained after electrodialysis was heated to 100 °C to allow the water and low-boiling-point organic acids present in the solution to volatilize. When the solution concentration reached 400 g/L, the solution was cooled to room temperature and then placed in a 10 °C water bath to produce precipitates. The precipitates were then redissolved in DI water to obtain a precipitate:water ratio of 1:12 (w/v), and the resultant solution was heated to 75 °C and incubated with 1% (w/v) activated carbon (TAC-TK, Taiwan Carbon Industry Co., Ltd.) for 60 min. After the removal of activated carbon from the solution through filtration, the solution was heated to 100 °C to volatilize the water present in it. When the solution concentration reached 350 g/L, the solution was cooled to room temperature and then placed in a 10 °C water bath to produce precipitates. Finally, the precipitates (crystals) were dried at 60 °C to obtain purified succinic acid.

### Analytical methods

The concentrations of succinate, ethanol, acetate, glycerol, and monosaccharides in the hydrolysate, fermentation broth, and solutions obtained in each separation or purification step were determined using a high-performance liquid chromatography pump (Hewlett Packard) equipped with an RI detector (RID-6A, Shimadzu). Sample mixtures were separated using a Bio-Rad Aminex HPX-87H column (300 × 7.8 mm^2^ i.d.) that was operated at 65 °C using a mobile phase of 5 mM H_2_SO_4_ at a flow rate of 0.6 mL/min. A standard curve for each analyte was made to quantify the concentration. Additional file 1: Figure S3 shows two typical HPLC spectra of fermentation broth composition.

## Supplementary Information

**Additional file 1: Figure S1.** PCR conditions, primers and DNA sequence alignment of alcohol dehydrogenase (Asuc_0403) in Mu-B7 mutant. “Query” is the DNA sequence of Asuc_0403 in the chromosome of *Actinobacillus succinogenes* 130Z (GenBank accession number CP000746.1), while “Sbjct” is the DNA sequence of Asuc_0403 in Mu-B7 mutant. **Figure S2.** Succinic acid fermentation on hydrolysate of glucose (a), xylose (b), and hydrolysate of Napier grass supplemented with glycerol at the mass ratio of 10:1 (c), 5:1 (d), and 2:1 (e). The ratios are based on the amount of glucose in hydrolysate to the amount of glycerol added, w/w. **Figure S3.** HPLC spectra of fermentation broth compositions: (a) fermentation on glucose for 8 h and (b) fermentation on the hydrolysate of Napier grass supplemented with glycerol at the hydrolysate-to-glycerol ratio of 2:1 for 8 h.

## Data Availability

The data sets used and/or analysed during the current study are available from the corresponding authors on reasonable request.
