# Supplementary material for: Production of succinic acid through the fermentation of Actinobacillus succinogenes on the hydrolysate of Napier grass
Source: Biotechnol Biofuels Bioprod. 2022 Jan 18;15:9. doi: 10.1186/s13068-022-02106-0 (PMC8767706; doi:10.1186/s13068-022-02106-0)
Supplement: Supplementary file 1 — Additional file 1: Figure S1. PCR conditions, primers and DNA sequence alignment of alcohol dehydrogenase (Asuc_0403) in Mu-B7 mutant. “Query” is the DNA sequence of Asuc_0403 in the chromosome of Actinobacillus succinogenes 130Z (GenBank accession number CP000746.1), while “Sbjct” is the DNA sequence of Asuc_0403 in Mu-B7 mutant. Figure S2. Succinic acid fermentation on hydrolysate of glucose (a), xylose (b), and hydrolysate of Napier grass supplemented with glycerol at the mass ratio of 10:1 (c), 5:1 (d), and 2:1 (e). The ratios are based on the amount of glucose in hydrolysate to the amount of glycerol added, w/w. Figure S3. HPLC spectra of fermentation broth compositions: (a) fermentation on glucose for 8 h and (b) fermentation on the hydrolysate of Napier grass supplemented with glycerol at the hydrolysate-to-glycerol ratio of 2:1 for 8 h. [file 13068_2022_2106_MOESM1_ESM.docx]

Production of succinic acid through the fermentation of *Actinobacillus succinogenes* on the hydrolysate of Napier grass

Jhih-Sing Lee, Cheng-Jia Lin, Wen-Chien Lee*, Hsin-Yi Teng, and Meng-Hsin Chuang

*Department of Chemical Engineering, Systems Biology and Tissue Engineering Research Center, National Chung Cheng University, Chiayi, Taiwan*

**Email: chmwcl@ccu.edu.tw*

**Additional Figures**

**PCR conditions:**

**∞**

**4 ℃**

**98℃**

**98℃**

**0:30**

**0:10**

**72℃**

**72℃**

**10:00**

**45s**

**56℃**

**30 s**

**30 cycles**

**Primers for PCR:**

| Forward primer: 5'-GAC GTA TTA ACG CAG GCG GA -3' |
| --- |
| Reverse primer: 5'-CGG CAT TTA CAA TCG GGG CG -3' |

**DNA Sequence alignment:**

| **Score** | **Expect** | **Identities** | **Gaps** | **Strand** |
| --- | --- | --- | --- | --- |
| 2139 bits (1158) | 0.0 | 1157/1158 (99%) | 0/1158 (0%) | Plus/Minus |

Query 1 ATGTCAACATATTATTTTTTACCAACCAGAAATGTATTCGGCGAAAATGCAGTTGAAGAA 60

||||||||||||||||||||||||||||||||||||||||||||||||||||||||||||

Sbjct 65 ATGTCAACATATTATTTTTTACCAACCAGAAATGTATTCGGCGAAAATGCAGTTGAAGAA 124

Query 61 GTCGGCACATTAATGAAAAGTTTAGGCGGCAACAATCCTCTGATTGTTACCGATGCTTTC 120

||||||||||||||||||||||||||||||||||||||| ||||||||||||||||||||

Sbjct 125 GTCGGCACATTAATGAAAAGTTTAGGCGGCAACAATCCTATGATTGTTACCGATGCTTTC 184

Query 121 CTCGCCAAAAACGGTATGGCGGATCAATTAGCCGCCGTATTAAGTAACGCAGGTTTAAAA 180

||||||||||||||||||||||||||||||||||||||||||||||||||||||||||||

Sbjct 185 CTCGCCAAAAACGGTATGGCGGATCAATTAGCCGCCGTATTAAGTAACGCAGGTTTAAAA 244

Query 181 CCGGTGATTTTCGGCGGTGCCGAACCGAATCCGACAGACAAAAACGTAGAAGAGGGTATT 240

||||||||||||||||||||||||||||||||||||||||||||||||||||||||||||

Sbjct 245 CCGGTGATTTTCGGCGGTGCCGAACCGAATCCGACAGACAAAAACGTAGAAGAGGGTATT 304

Query 241 GTGTTTTATAACGAACACGGTTGCGATTCCATCATTTCTTTGGGCGGTGGTTCCTCCCAC 300

||||||||||||||||||||||||||||||||||||||||||||||||||||||||||||

Sbjct 305 GTGTTTTATAACGAACACGGTTGCGATTCCATCATTTCTTTGGGCGGTGGTTCCTCCCAC 364

Query 301 GACTGTGCCAAAGGTATCGGTTTAATCGCCAGTAACGGCGGACGCATTCAGGATTACGAA 360

||||||||||||||||||||||||||||||||||||||||||||||||||||||||||||

Sbjct 365 GACTGTGCCAAAGGTATCGGTTTAATCGCCAGTAACGGCGGACGCATTCAGGATTACGAA 424

Query 361 GGCGTCGATCGTTCCCACAATGCGATGGTACCGCTGATGGCGGTCAACACCACGGCGGGA 420

||||||||||||||||||||||||||||||||||||||||||||||||||||||||||||

Sbjct 425 GGCGTCGATCGTTCCCACAATGCGATGGTACCGCTGATGGCGGTCAACACCACGGCGGGA 484

Query 421 ACGGCGTCTGAAATCACCCGGTTCTGTATTATTACCGACACGGCGCGGAAAGTGAAAATG 480

||||||||||||||||||||||||||||||||||||||||||||||||||||||||||||

Sbjct 485 ACGGCGTCTGAAATCACCCGGTTCTGTATTATTACCGACACGGCGCGGAAAGTGAAAATG 544

Query 481 GCGATTGTAGACTGGCGCATCACTCCGCAAATCGCGGTGAACGATCCATTGTTGATGAAA 540

||||||||||||||||||||||||||||||||||||||||||||||||||||||||||||

Sbjct 545 GCGATTGTAGACTGGCGCATCACTCCGCAAATCGCGGTGAACGATCCATTGTTGATGAAA 604

Query 541 GGCATGCCGCCAAGCCTGACCGCAGCAACCGGTATGGACGCCCTGACGCACGCAATCGAA 600

||||||||||||||||||||||||||||||||||||||||||||||||||||||||||||

Sbjct 605 GGCATGCCGCCAAGCCTGACCGCAGCAACCGGTATGGACGCCCTGACGCACGCAATCGAA 664

Query 601 GCCTACGTATCCACTGCCGCCAACCCGCTCACGGATGCCGCAGCGCTGATGGCGATCACC 660

||||||||||||||||||||||||||||||||||||||||||||||||||||||||||||

Sbjct 665 GCCTACGTATCCACTGCCGCCAACCCGCTCACGGATGCCGCAGCGCTGATGGCGATCACC 724

Query 661 ATGATTCAGCAATACCTGCCGAAAGCGGTAGCGAACGGCGACTATATGAAAGCCCGCGAT 720

||||||||||||||||||||||||||||||||||||||||||||||||||||||||||||

Sbjct 725 ATGATTCAGCAATACCTGCCGAAAGCGGTAGCGAACGGCGACTATATGAAAGCCCGCGAT 784

Query 721 AAAATGGCATATGCGCAATATTTGGCAGGTATCGCTTTCAATAATGCCTCTCTCGGCTAT 780

||||||||||||||||||||||||||||||||||||||||||||||||||||||||||||

Sbjct 785 AAAATGGCATATGCGCAATATTTGGCAGGTATCGCTTTCAATAATGCCTCTCTCGGCTAT 844

Query 781 GTTCATGCGATGGCACACCAACTGGGCGGTTTCTACAACCTGCCGCACGGTGTGTGTAAC 840

||||||||||||||||||||||||||||||||||||||||||||||||||||||||||||

Sbjct 845 GTTCATGCGATGGCACACCAACTGGGCGGTTTCTACAACCTGCCGCACGGTGTGTGTAAC 904

Query 841 GCGATTCTGTTGCCTTATGTGGAAGAATTCAACCTCATCGGTAATCTCAACCGTTTCCGC 900

||||||||||||||||||||||||||||||||||||||||||||||||||||||||||||

Sbjct 905 GCGATTCTGTTGCCTTATGTGGAAGAATTCAACCTCATCGGTAATCTCAACCGTTTCCGC 964

Query 901 GACATCGCCAAAGCTATGGGTGAAAATATCGACGGTTTATGCACGGACGATGCGGCACTG 960

||||||||||||||||||||||||||||||||||||||||||||||||||||||||||||

Sbjct 965 GACATCGCCAAAGCTATGGGTGAAAATATCGACGGTTTATGCACGGACGATGCGGCACTG 1024

Query 961 AAAGCCATTGGCGCGATTCGCCGTTTAAGCAAACAAGTGGGAATCCCGGCAAATCTGCAA 1020

||||||||||||||||||||||||||||||||||||||||||||||||||||||||||||

Sbjct 1025 AAAGCCATTGGCGCGATTCGCCGTTTAAGCAAACAAGTGGGAATCCCGGCAAATCTGCAA 1084

Query 1021 CTGCTCGGCGTGAAACCGGAAGATTTCGACGTGATGGCGGAAAATGCGATGAAAGACGTG 1080

||||||||||||||||||||||||||||||||||||||||||||||||||||||||||||

Sbjct 1085 CTGCTCGGCGTGAAACCGGAAGATTTCGACGTGATGGCGGAAAATGCGATGAAAGACGTG 1144

Query 1081 TGTATGCTCACCAATCCGCGCAAAGCCACCAAACAGCAAGTCATCGAAATTTTCCAACGG 1140

||||||||||||||||||||||||||||||||||||||||||||||||||||||||||||

Sbjct 1145 TGTATGCTCACCAATCCGCGCAAAGCCACCAAACAGCAAGTCATCGAAATTTTCCAACGG 1204

Query 1141 GCTTATGACGGCGATTAA 1158

||||||||||||||||||

Sbjct 1205 GCTTATGACGGCGATTAA 1222

Figure S1. PCR conditions, primers and DNA sequence alignment of alcohol dehydrogenase (Asuc_0403) in Mu-B7 mutant. “Query” is the DNA sequence of Asuc_0403 in the chromosome of *Actinobacillus succinogenes*130Z (GenBank accession number CP000746.1), while “Sbjct” is the DNA sequence of Asuc_0403 in Mu-B7 mutant.


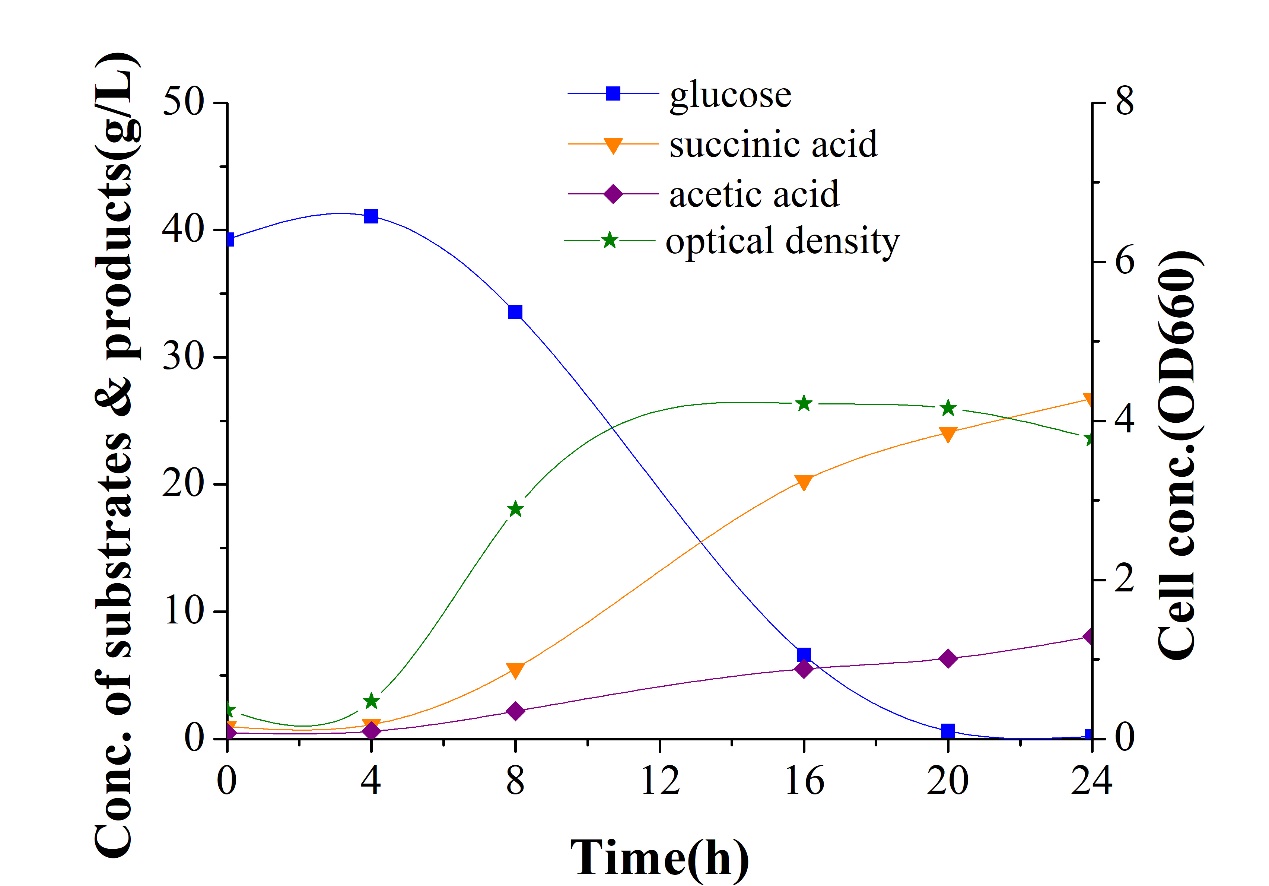


(a)


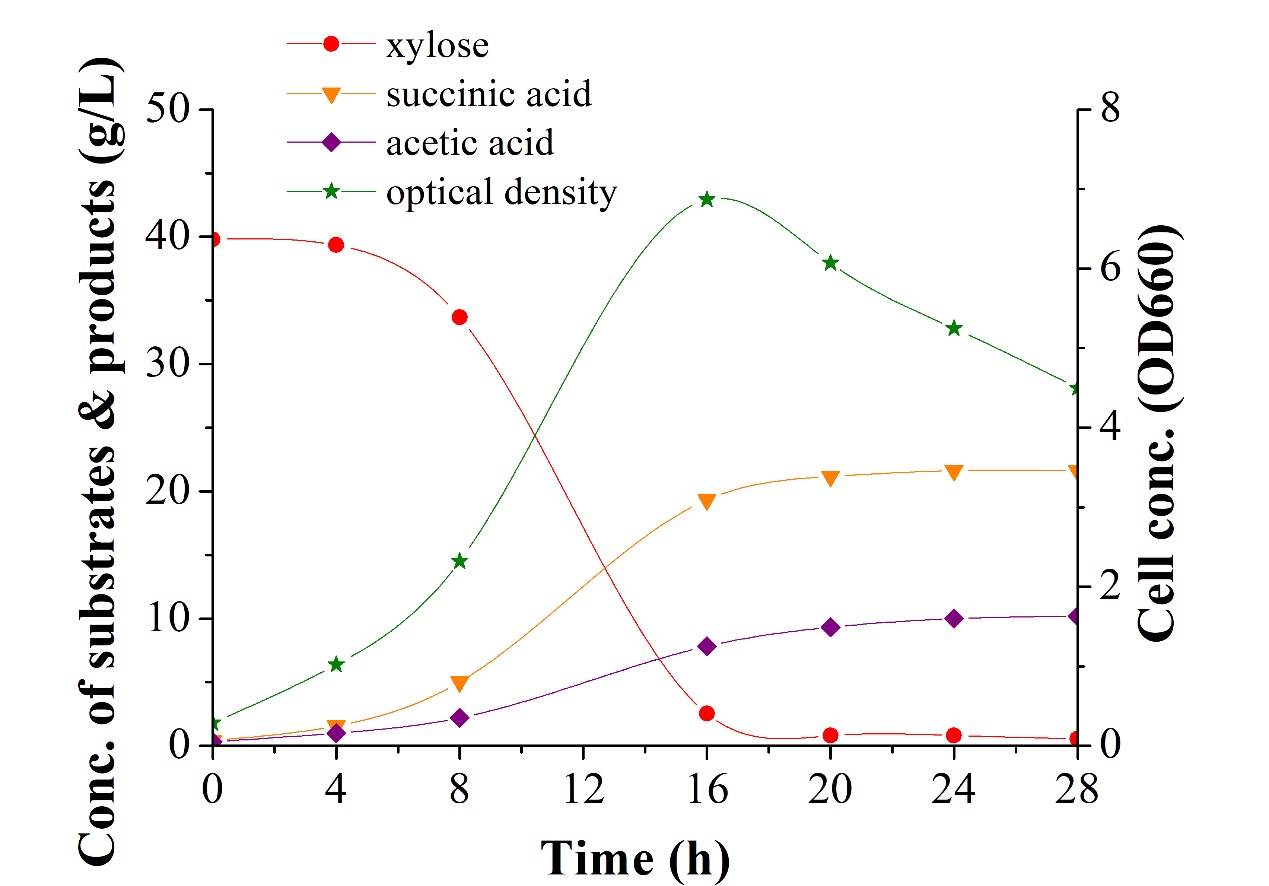


(b)


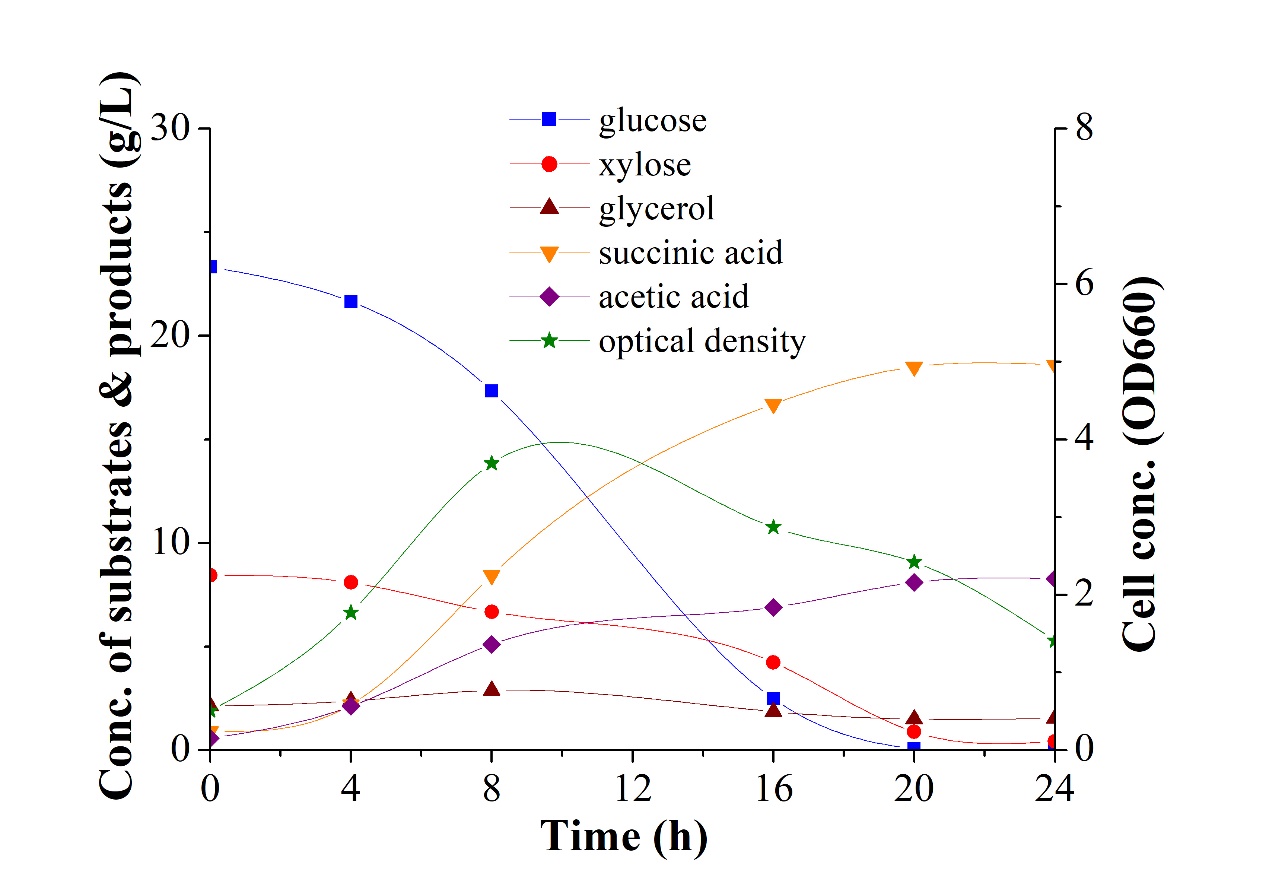


(c)


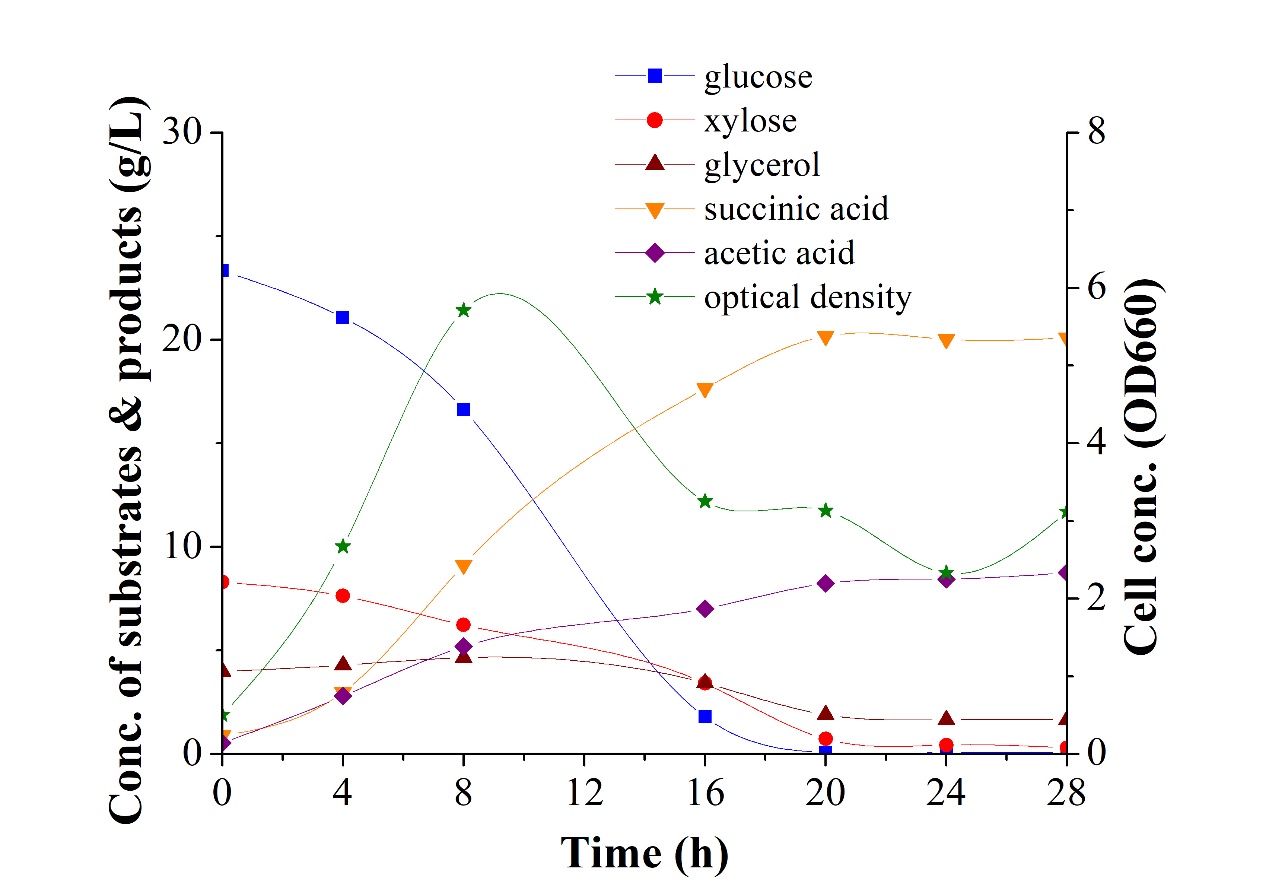


(d)


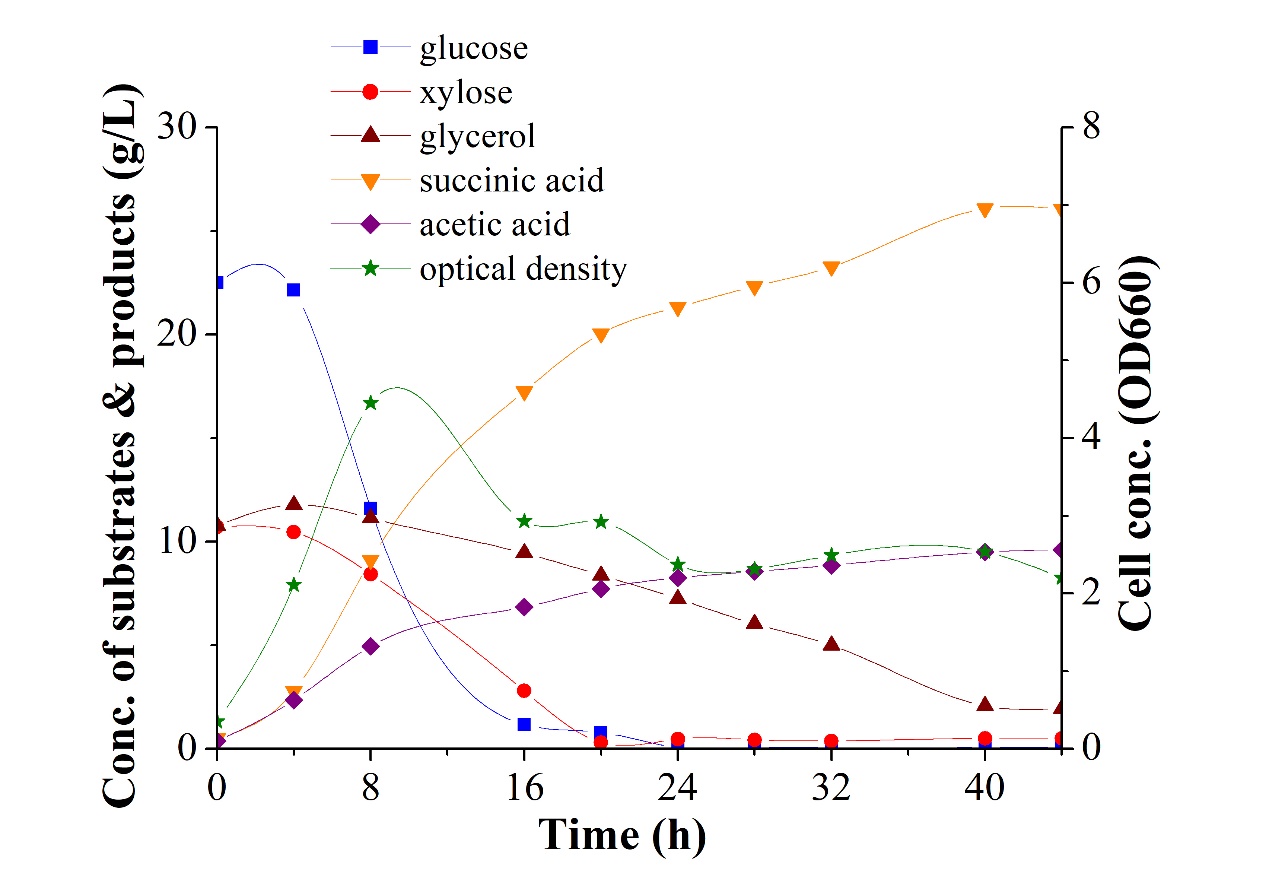


(e)

Figure S2. Succinic acid fermentation on hydrolysate of glucose (a), xylose (b), and hydrolysate of Napier grass supplemented with glycerol at the mass ratio of 10:1 (c), 5:1 (d), and 2:1 (e). The ratios are based on the amount of glucose in hydrolysate to the amount of glycerol added, w/w.


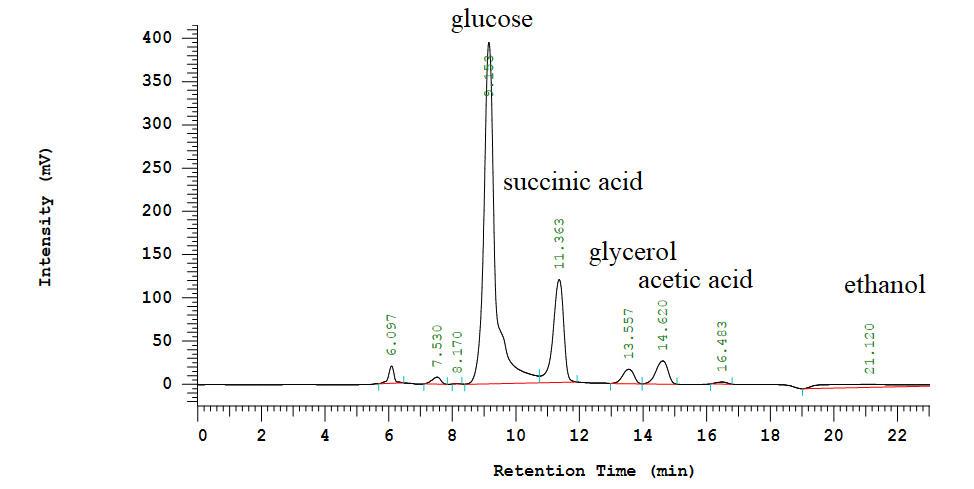


(a)


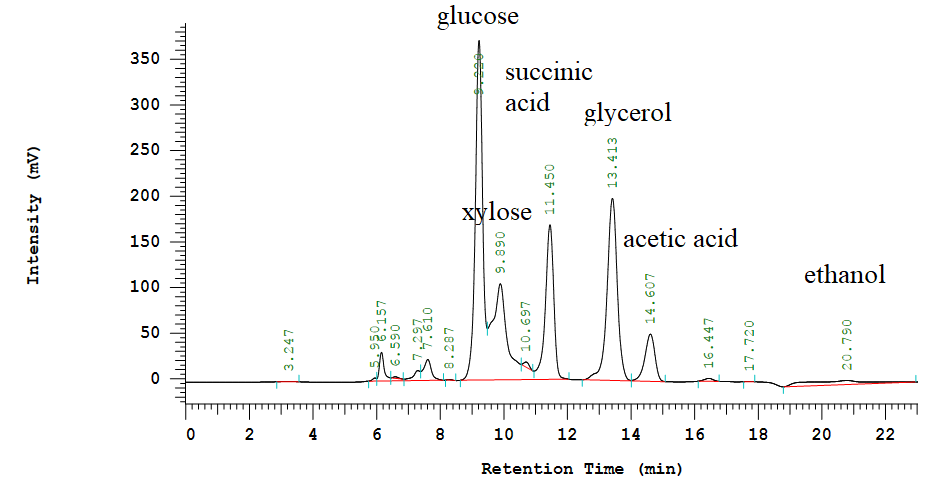


(b)

Figure S3. HPLC spectra of fermentation broth compositions: (a) fermentation on glucose for 8h and (b) fermentation on the hydrolysate of Napier grass supplemented with glycerol at the hydrolysate-to-glycerol ratio of 2:1 for 8 h.
